# Performance of postmortem CT in the diagnosis of natural death from out-of-hospital cardiac arrest

**DOI:** 10.1007/s11604-024-01559-7

**Published:** 2024-04-16

**Authors:** Yu Nakaki, Wataru Fukumoto, Haruka Higashibori, Ikuo Kawashita, Yuko Nakamura, Kazuo Awai

**Affiliations:** 1 Department of Diagnostic Radiology, Graduate School of Biomedical and Health Science, Hiroshima University, 1-2-3 Kasumi, Minamiku, Hiroshima, 734-8551 Japan; 2 Center for Cause of Death Investigation Research, Graduate School of Biomedical and Health Science, Hiroshima University, 1-2-3 Kasumi, Minamiku, Hiroshima, 734-8551 Japan; 3 Department of Diagnostic Imaging, JA Hiroshima General Hospital, 1-3-3 Jigozen, Hatsukaichi-Shi, Hiroshima, 738-0042 Japan

**Keywords:** Postmortem CT, Postmortem imaging, Natural death, Out-of-hospital cardiac arrest

## Abstract

**Purpose:**

Postmortem CT (PMCT) is used widely to identify the cause of death. However, its diagnostic performance in cases of natural death from out-of-hospital cardiac arrest (OHCA) may be unsatisfactory because the cause tends to be cardiogenic and cannot be detected on PMCT images. We retrospectively investigated the diagnostic performance of PMCT in the diagnosis of natural death from OHCA and compared it to that of unnatural death.

**Materials and methods:**

Our series included 450 cases; 336 were natural- and 114 were unnatural death cases. Between 2018 and 2022 all underwent non-contrast PMCT to identify the cause of death. Two radiologists reviewed the PMCT images and categorized them as diagnostic (PMCT alone sufficient to determine the cause of death), suggestive (the cause of death was suggested but additional information was needed), and non-diagnostic (the cause of death could not be determined on PMCT images). The diagnostic performance of PMCT was defined by the percentage of diagnosable and suggestive cases and compared between natural- and unnatural death cases. Interobserver agreement for the cause of death on PMCT images was also assessed with the Cohen kappa coefficient of concordance.

**Results:**

The diagnostic performance of PMCT for the cause of natural- and unnatural deaths from OHCA was 30.3% and 66.6%, respectively (p < 0.01). The interobserver agreement for the cause of natural- and unnatural deaths on PMCT images was very good with kappa value 0.92 and 0.96, respectively.

**Conclusion:**

As PMCT identified the cause of natural death by OHCA in only 30% of cases, its diagnostic performance must be improved.

## Introduction

The accurate identification of the cause of death is essential for the acquisition of epidemiological data, for treatment quality control, for the re-evaluation of diagnostic and therapeutic management, and for clinical and medical education [1, 2]. Although conventional autopsy is a traditional procedure to identify the cause of death, there has been a marked decline in autopsy rates worldwide recently, with autopsy rates reaching less than 10% of deaths in many countries due to a lack of forensic pathologists, the risk of infection, high costs, and religious proscriptions [1, 3]. Consequently, postmortem CT (PMCT) has become a complementary tool for conventional autopsy [4–8].

Although PMCT can help to identify the cause of death, the diagnostic performance of PMCT is affected by the condition of the corpse and the applied methodology. In cases of unnatural death, PMCT can reveal traumatic changes such as bone fractures, organic injuries, and hemorrhages [9–13]. The diagnostic performance of PMCT in unnatural deaths is relatively high at 46–100% [4, 6, 11, 13]. For in-hospital deaths, PMCT is useful for establishing the cause of death in 65–75% of cases because the interval between death and PMCT examination is short, and antemortem information such as clinical records, laboratory findings, and final antemortem CT imaging is available [5, 14].

On the other hand, the diagnostic performance of PMCT in natural deaths is only 40–50% [6, 8] because myocardial infarction, lethal arrhythmia, pulmonary thromboembolism, and pneumonia are frequently missed on non-contrast PMCT images [7]. Especially in cases of natural death from out-of-hospital cardiac arrest (OHCA), its performance may be unsatisfactory because the cause of OHCA tends to be cardiogenic [15–17] and antemortem information is often insufficient. However, few studies investigated the diagnostic performance of PMCT in cases of natural death from OHCA.

To reveal the current issue of PMCT, we retrospectively investigated the diagnostic performance of PMCT in the identification of the cause of natural death from OHCA and compared it to that of unnatural death.

## Materials and methods

This retrospective study was approved by our institutional review board; prior informed consent was waived.

### Subjects

For this study of the diagnostic performance of PMCT we initially selected 512 OHCA cases that we encountered in the emergency department at our hospital between January 2018 and December 2022. All underwent non-contrast PMCT; 15 cases younger than 18 years were subsequently dropped. We also excluded 47 that were bath-related deaths because it was difficult to determine whether the cause of death was natural or unnatural. At the time of PMCT scanning, of the 450 remaining cases, 336 were considered natural- and 114 unnatural deaths. Whether death was natural or unnatural was determined by emergency physicians based on body-surface observations and information obtained from the paramedics, family members, or persons who found the body. A natural death results from a disease or aging, while an unnatural death results from external causes such as accidents, suicide, drowning and drug abuse. Of the natural death cases, 181 were of males, 155 were of females; the median age was 79 years (range 18–104 years). Of the 114 unnatural death cases, 65 were male- and 49 were female cases; the median age was 57 years (range 19–99 years). Of 450 OHCA cases, 361 cases were new patients and adequate medical histories were not available. The other 89 patients had visited our hospital for malignant tumors (n = 32), cardiovascular disease (n = 26), respiratory disease (n = 9), cerebrovascular disease (n = 5), liver disease (n = 5) and other diseases (n = 12).

### PMCT scanning

PMCT was on a 320-row multi-detector scanner (Aquilion One; Canon Medical Systems). Helical scans were acquired at a tube voltage of 120 kV; the tube current was regulated by automatic exposure control. The preset noise level was 12–13 Hounsfield units (HU) for the body and 3 HU for the head, the slice thickness was 5 mm. The scanning parameters were rotation time 0.5 s (body) and 1.0 s (head), beam collimation 0.5 mm × 320, helical pitch 1.38 (body) and 0.83 (head), display field-of-view 50 cm (body) and 35 cm (head).

The interval between death and PMCT was defined as the time that elapsed between the time cardiac arrest was noticed by witnesses and the time the body underwent PMCT. If there were no witnesses, it was defined as the time when the person was last seen alive to PMCT scanning. The median of interval between death and PMCT was 2 h (range 1–28 h).

### Image interpretation

Two board-certified radiologists (W.F. and H.H) with 14 and 9 years of experience whose specialty was forensic medicine independently interpreted the PMCT images (axial images, slice thickness 5 mm, multi-planar reconstruction images were acquired when necessary), with kernels for soft tissue and lung. The readers had simple clinical information related to the study subjects’ medical history, the circumstances at the time of cardiac arrest, and the external findings. Laboratory findings such as blood, urine and cerebrospinal fluid were not provided because such findings are sometimes insufficient in OHCA cases and our study focused on the diagnostic performance of PMCT. The readers identified the immediate cause of death on PMCT images by referring to earlier reports [6, 8] and recorded the images consensually as diagnostic (PMCT alone was sufficient to determine the cause of death), suggestive (the cause of death was suggested but additional information was needed), and non-diagnostic (the cause of death could not be determined on the PMCT images).

### Statistical analysis

Interobserver agreement for the cause of death on PMCT images was assessed with the Cohen kappa coefficient of concordance. Kappa values of 0–0.20 indicated poor-, 0.21–0.40 fair-, 41–0.60 moderate-, 0.61–0.80 good-, and > 0.81 very good agreement.

The diagnostic performance of PMCT was defined as the percentage of diagnosable and suggestive cases and compared between natural- and unnatural deaths using a Chi-square test.

Differences of p < 0.05 were considered statistically significant. We used software for statistical analysis (JMP software, SAS Institute).

## Results

**Fig. 1:**
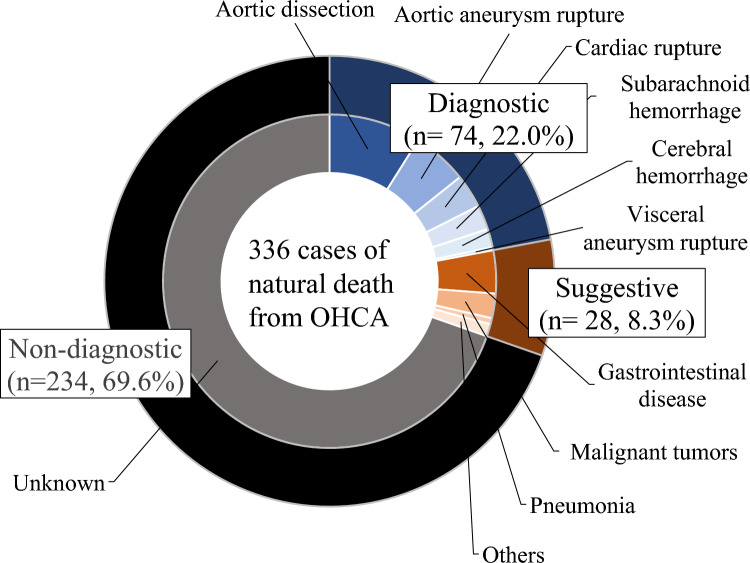
The distribution of PMCT images of natural deaths from OHCA categorized as diagnostic-, suggestive- and non-diagnosable. Aortic dissection, aortic aneurysm rupture, cardiac rupture, subarachnoid hemorrhage, cerebral hemorrhage, and visceral aneurysm rupture were diagnosable. In the suggestive category were gastrointestinal disease, malignant tumors, pneumonia, and other causes

Of the 336 PMCT images of natural death cases, 74 (22.0%) were diagnostic, 28 (8.3%) were suggestive, and 234 (69.6%) were non-diagnostic (Fig. 1). Diagnosable were aortic dissection (n = 30), aortic aneurysm rupture (n = 18), cardiac rupture (n = 11), subarachnoid hemorrhage (n = 8), cerebral hemorrhage (n = 6) and visceral aneurysm rupture (n = 1). Gastrointestinal disease (n = 14) [e.g. bleeding, occlusion and perforation], malignant tumors (n = 8), pneumonia (n = 2), and 4 other causes were suggestive. The interobserver agreement was very good [kappa value 0.92 (95% confidence interval (CI) = 0.88–0.97)]; 74/74 (100%) in diagnosable cases, 26/28 (92.9%) in suggestive cases and 224/234 (95.7%) in non-diagnostic cases were agreed between readers. With respect to 2 suggestive cases, one reader judged that pneumonia was the cause of death while the other reader was unable to ascertain that it was the cause of death. There was no reader agreement in 10 non-diagnosable natural death cases (pneumonia n = 8, gastrointestinal obstruction (n = 1), malignant tumor (n = 1).

**Fig. 2:**
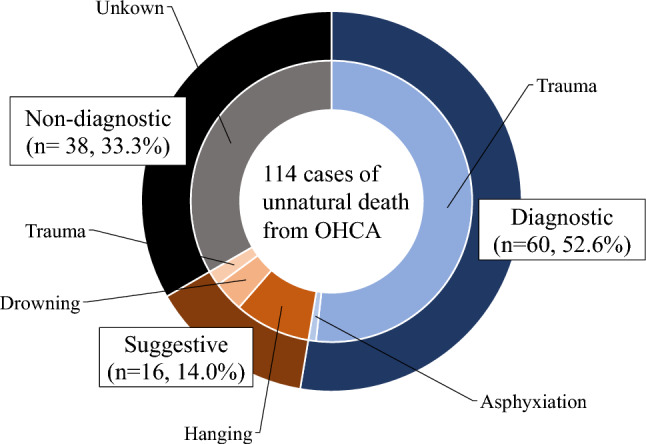
The distribution of PMCT images of unnatural deaths categorized as diagnostic, suggestive, and non-diagnosable. Trauma and asphyxiation were diagnosable. Suggestive images included hanging, drowning, and lethal trauma

Of the 114 PMCT images of unnatural death cases, 60 (52.6%) were diagnostic, 16 (14.0%) were suggestive, and 38 (33.3%) were non-diagnostic (Fig. 2). Lethal trauma (n = 59) and asphyxiation (n = 1) were diagnosable. Suggestive images included deaths from hanging (n = 10), drowning (n = 4) and trauma (n = 2). The interobserver agreement was very good [kappa value 0.96 (95% CI = 0.91—1.00)]. Both readers agreed in 60/60 (100%) diagnosable cases, 14/16 (87.5%) suggestive cases, and 37/38 (97.4%) non-diagnostic cases. The readers were divided on whether two drownings in suggestive cases and one pneumothorax among the non-diagnosable cases were the cause of death.

The diagnostic performance of PMCT for the cause of natural and unnatural death was 30.3% and 66.6%, respectively; its performance for natural death was significantly lower than that of for unnatural death (p < 0.01).

**Fig. 3:**
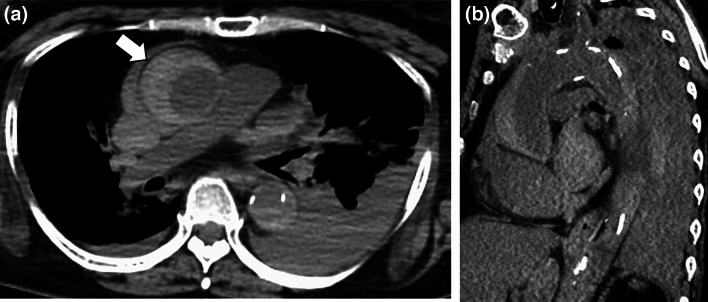
A 63-year-old woman in cardiac arrest who was found at home. Axial PMCT showed a highly attenuated false lumen (arrow) in the ascending aorta reflecting dissection (**a**). The oblique sagittal image revealed aortic dissection from the ascending- to the descending aorta (**b**). PMCT diagnosed aortic dissection as the cause of death

**Fig. 4:**
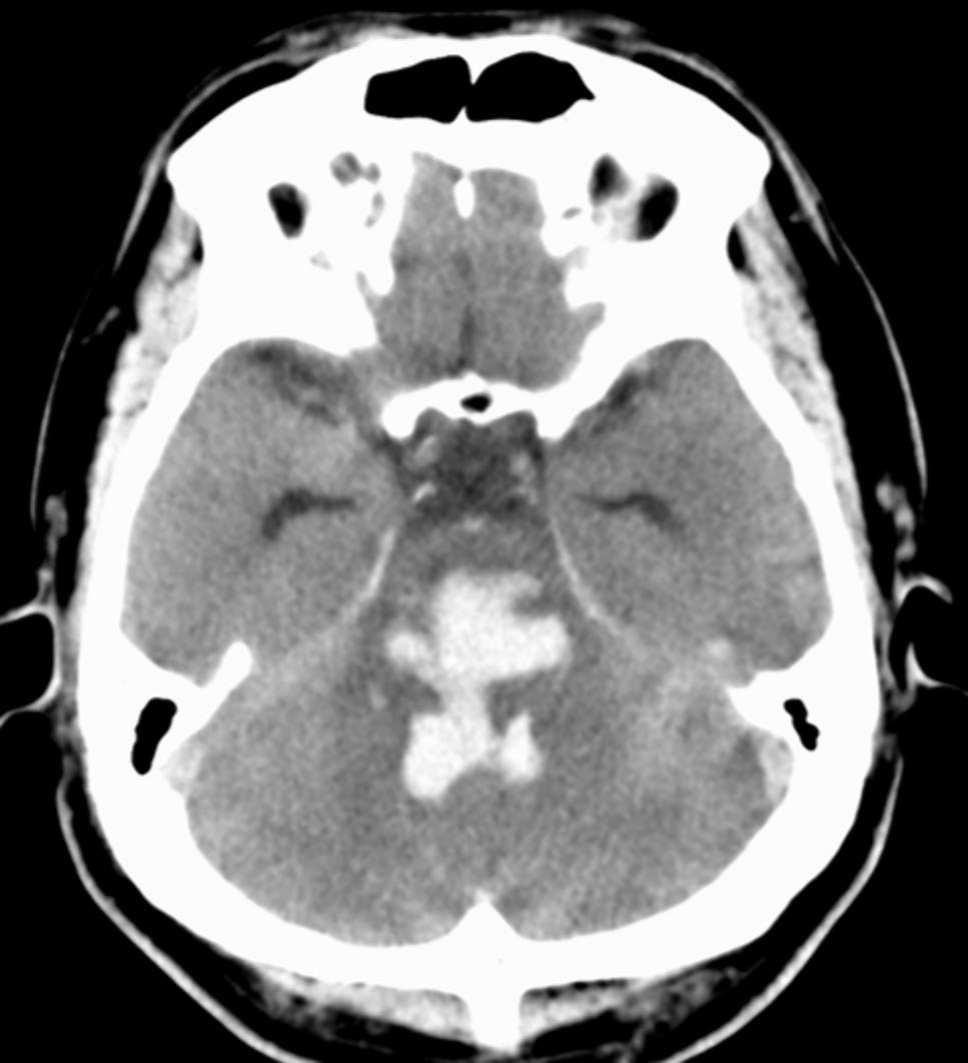
A 46-year-old man in cardiac arrest who was found at home. Axial PMCT showed hemorrhage in the brainstem and the 4th ventricle as a highly attenuated mass. PMCT diagnosed cerebral hemorrhage as the cause of death

**Fig. 5:**
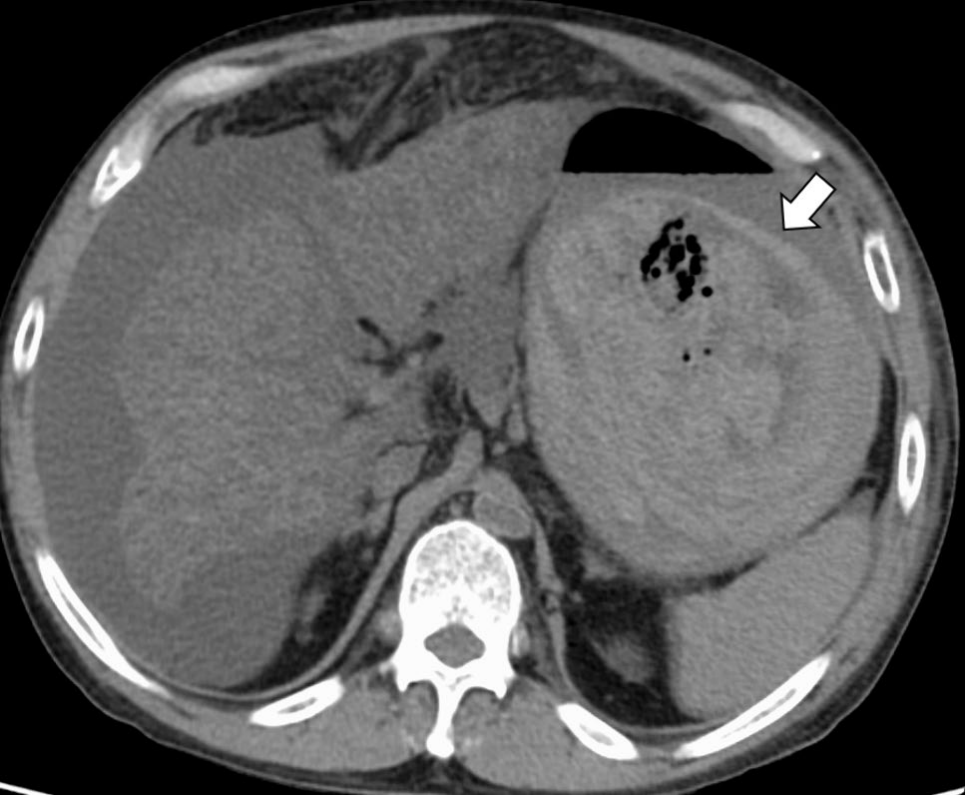
A 55-year-old man vomited blood at home and went into cardiac arrest. Axial PMCT showed a large hematoma in the stomach as a highly attenuated area (arrows). Liver cirrhosis and ascites are also seen. PMCT suggested gastrointestinal bleeding as the cause of death

**Fig. 6:**
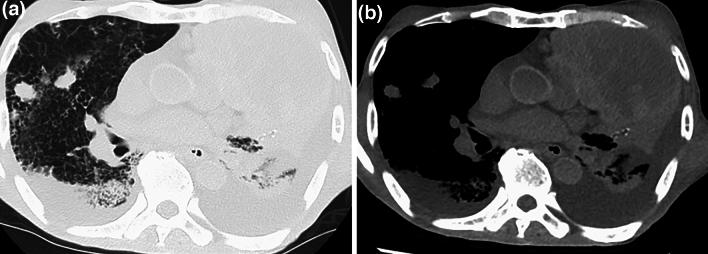
A 79-year-old man treated for lung cancer. He went into cardiac arrest at home after breathing difficulties. Axial PMCT showed lung cancer in the left upper lobe and many metastatic nodules in the right lung (**a**). Extensive bilateral pleural effusion was also observed (**b**). PMCT suggested multiple metastases from lung cancer as the cause of death

Representative cases of natural death from OHCA are presented in Figs. 3, 4, 5, 6.

## Discussion

We report that the diagnostic performance of PMCT for identifying the cause of natural death from OHCA was significantly lower than for unnatural death (30.3% vs 66.6%, p < 0.01). PMCT could estimate the immediate cause of death in approximately 30% of natural deaths from intracranial hemorrhage, aortic dissection, aortic aneurysm rupture, cardiac rupture, gastrointestinal disease, malignant tumors, and pneumonia. These diseases account for about 30% of natural deaths from OHCA seen in emergency departments. In such cases, PMCT would be useful for determining the cause of death and revealing it to the bereaved family. On the other hand, PMCT was not useful in 70% of natural deaths from OHCA and its diagnostic usefulness in such cases remains too low.

OHCA is a major global public health issue. According to the Japanese Fire and Disaster Management Agency, more than 120,000 Japanese OHCA cases are reported annually [16]. Although most OHCA patients cannot be rescued, the surviving families often demand to know the cause of death and this information is also needed for the development of medicine. Because a majority of OHCAs are due to primary cardiogenic diseases such as myocardial infarction and lethal arrythmia [15–17], they are non-diagnosable on non-contrast PMCT images [6, 8]. Besides, it is difficult to distinguish antemortem pulmonary diseases such as pneumonia and cardiogenic pulmonary edema from postmortem changes or pulmonary edema due to other causes on PMCT images [6, 7, 18] because antemortem information is often insufficient in deaths from OHCA. In fact, with respect to a diagnosis of pneumonia as the cause of death in 10 cases, the readers were not in agreement. Consequently, the diagnostic performance of PMCT for identifying the cause of natural death from OHCA is unsatisfactory.

To date, few studies investigated the diagnostic performance of PMCT in cases of natural death from OHCA. As did we, Kaneko et al. [19] found that it was useful for establishing the cause of death in only 30% of cases with sudden unexpected natural death. In 38.1% of non-traumatic deaths, Takahashi et al. [8] detected the fatal findings such as intracranial hemorrhage, aortic dissection and aortic aneurysm rapture on PMCT images; however, their cases included deaths from drowning, asphyxiation, and initially unsuspected trauma. Although PMCT revealed that the cause of natural death was identified in 52.0% of 65 forensic cases reported by Kasahara et al. [6]; they did not focus on OHCA deaths and they recorded all pneumonia cases as suggestive. We, on the other hand, categorized only two PMCT images of pneumonia as suggestive because antemortem information was scarce. The cited earlier reports were published more than 10 years ago and our current findings indicate that the diagnostic performance of PMCT has not improved sufficiently. Additional information about the elemental chemical composition of material scanned by CT can be obtained with dual-energy CT and photon-counting detector CT; their availability may represent a breakthrough for the improvement of the diagnostic performance of PMCT [20, 21]. Using these advanced CT scanners, myocardial edema due to myocardial infarcts and pulmonary embolism may be diagnosable but further investigations are necessary.

The value of PMCT angiography (PMCTA) to diagnose coronary artery stenosis and pulmonary embolism has been investigated [22, 23]. Westphal et al. [22] reported that its diagnostic accuracy in cases of cardiac death was 80%. Others [7, 24] documented that postmortem MRI (PMMRI) detected myocardial infarcts and pulmonary embolism. According to Roberts et al. [7], in combination, PMCT and PMMRI improved identifying the cause of natural death by 14% compared to PMCT alone; in 70% of cases, the postmortem imaging- and autopsy findings were consistent.

To diagnose the cause of death, minimally invasive autopsies (MIA) apply a needle-based approach and ultrasound, CT, or endoscopic guidance to collect samples from key organs without opening the cadaver [25, 26]. Since MIA can diagnose cardio-pulmonary disease, e.g. myocardial infarction, pneumonia, pulmonary edema and adult distress respiratory syndrome histologically, combined PMCT, PMCTA and MIA yielded 90.9% sensitivity for the cause of death [25]. If myocardial infarction or pneumonia is clinically suspected, adding MIA at the time of PMCT scanning may provide a more accurate diagnosis of the cause of death. Despite its success, this technique is rarely applied due to its complexity, cost, and the lack of trained specialists [26, 27]. The application of these techniques and the training of specialists are future challenges.

Although this study focused on the diagnostic performance of PMCT for identifying the immediate cause of death in OHCA cases, a careful observation of the body surface, understanding of the surrounding circumstances at the time of the death, and various laboratory findings such as blood-, urine-, cerebrospinal fluid-, and drug results are also essential for an accurate and specific identification of the cause of death. It is also important to understand the limitations and pitfalls of postmortem imaging, and to perform autopsy when the involvement of an incident or in a crime cannot be ruled out.

Our retrospective study has some limitations. It was conducted at a single institution, and the diagnostic performance of PMCT may vary at different institutions. Nonetheless, as ours is a representative tertiary emergency care facility, we consider our findings relevant. Our subjects were OHCA patients seen in the emergency department and antemortem information such as the circumstances under which the body was found, and the time elapsed since death was insufficient in some cases. Therefore, not all of the natural- or unnatural death classifications determined by emergency physicians may have been correct. However, it is important to point out that none of our death classifications as “natural” or “unnatural” death were later rescinded. We did not compare PMCT- and autopsy findings because most included cadavers were not autopsied. In our study we referred to findings reported by others [6, 8] who have provided sufficient evidence of PMCT-diagnosable diseases. Although we could not present a direct correlation between PMCT- and autopsy findings, we concluded that the diagnostic performance of PMCT in actual OHCA cases was satisfactory. Lastly, our interpretation of the PMCT images was subjective and it may reflect the readers’ experience in the interpretation of such images especially in suggestive cases. Therefore, the readers did not agree as to the cause of death in some cases (pneumonia, gastrointestinal obstruction, malignancy, and drowning). Nonetheless, our readers specialized in the interpretation of PMCT images and the interobserver agreement was very good even in suggestive cases. The readers charged with the interpretation of PMCT images require specialized training.

In conclusion, the current diagnostic performance of PMCT for identifying the cause of natural death from OHCA was only 30.3%.

## Abbreviations

PMCT: Postmortem computed tomography
OHCA: Out-of-hospital cardiac arrest
CI: Confidence interval
PMMRI: Postmortem MRI
PMCTA: Postmortem computed tomography angiography
MIA: Minimal invasive autopsy
